# Insights into pathophysiology and therapeutic strategies for heat stroke: Lessons from a baboon model

**DOI:** 10.1113/EP091586

**Published:** 2023-12-20

**Authors:** Mashan Abdullah, Salleh Ehaideb, George Roberts, Abderrezak Bouchama

**Affiliations:** ^1^ Experimental Medicine Department, King Abdullah International Medical Research Center/King Saud bin Abdulaziz University for Health Sciences, King Abdulaziz Medical City Ministry of National Guard Health Affairs Riyadh Saudi Arabia; ^2^ Pathology and Laboratory Medicine King Faisal Specialist Hospital and Research Center Riyadh Saudi Arabia

**Keywords:** cell death, coagulation abnormalities, heat stress response, hyperthermia, inflammatory response, organ injury

## Abstract

Heat stroke is a perilous condition marked by severe hyperthermia and extensive multiorgan dysfunction, posing a considerable risk of mortality if not promptly identified and treated. Furthermore, the complex biological mechanisms underlying heat stroke‐induced tissue and cell damage across organ systems remain incompletely understood. This knowledge gap has hindered the advancement of effective preventive and therapeutic strategies against this condition. In this narrative review, we synthesize key insights gained over a decade using a translational baboon model of heat stroke. By replicating heat stroke pathology in a non‐human primate species that closely resembles humans, we have unveiled novel insights into the pathways of organ injury and cell death elicited by this condition. Here, we contextualize and integrate the lessons learned concerning heat stroke pathophysiology and recovery, areas that are inherently challenging to investigate directly in human subjects. We suggest novel research directions to advance the understanding of the complex mechanisms underlying cell death and organ injury. This may lead to precise therapeutic strategies that benefit individuals suffering from this debilitating condition.

## Abstract (highlights)

- **What is the topic of this review?**
  The review focuses on understanding the pathophysiology of heat stroke using a translational baboon model.
- **What advances does it highlight?**
  The baboon model has unveiled intricate mechanisms of inflammatory, coagulation and cell death processes during heat stroke, and tested therapeutic interventions. Treatments targeting individual pathways were found ineffective, highlighting the complexity of heat stroke pathophysiology.

## 1 INTRODUCTION

Heat stroke is a critical medical emergency characterized by central nervous system alterations associated with a rapid increase in core body temperature to often over 40°C. This condition can occur upon exposure to high ambient temperatures (classic or non‐exertional heat stroke) or during strenuous exercise (exertional heat stroke). Heat stroke can rapidly progress to multiorgan injury, and without prompt diagnosic and immediate cooling, it can be fatal, with the central nervous system being particularly vulnerable (Bouchama et al., 2022).

Climate change‐driven heat waves are increasing the risk of classic heat stroke, posing a significant clinical challenge (Mora et al., 2017). Despite advancements of understanding its pathophysiology, mortality and morbidity rates associated with classic heat stroke remain high (Argaud et al., 2007; Dematte et al., 1998; Misset et al., 2006). In intensive care unit settings, classic heat stroke mortality ranges from 21% to 62.6%, even with optimal care, and survivors often endure severe neurological damage, contributing to long‐term mortality (Argaud et al., 2007; Dematte et al., 1998; Misset et al., 2006). Specific therapies beyond cooling are currently lacking, highlighting the urgent need for a deeper mechanistic understanding and the development of effective treatments (Bouchama et al., 2022). Consequently, we developed a non‐human primate (NHP) model of classic heat stroke to investigate the mechanisms of multiorgan injury and cell death, with the aim of identifying therapies to complement cooling.

NHP models have proven invaluable for gaining insights into complex conditions such as sepsis, elucidating aspects of tissue injury, inflammation, coagulation abnormalities, and cell death (Taylor et al., 2012; VandeBerg et al., 2009). NHPs, especially baboons, offer a suitable model for exploring the pathophysiology of heat stroke (Gathiram, Gaffin, et al., 1987; Hales et al., 1979). In innate immunity and coagulation system, NHPs are the closest to humans among animal species, making them particularly critical in the study of heat stroke, where these pathways play a significant role (Abildgaard et al., 1971; McFarlane et al., 2011). This similarity allows for the unraveling of intricate mechanisms of inflammation and coagulation in heat stroke and the testing of countermeasures that can be directly extrapolated to human conditions. The cardiovascular and thermoregulatory responses of NHPs to moderate heat stress closely resemble those of humans more than any other species (Hales et al., 1979). Moreover, NHPs subjected to extreme heat develop heat stroke replicating the clinical and haemodynamic alterations observed in human heat stroke (Gathiram, Gaffin, et al., 1987; Gathiram et al., 1988).

Over a decade (2000–2010), we used the baboon model to investigate heat stroke pathophysiology, as documented in our series of studies (Bouchama, Ollivier, et al., 2005; Bouchama, Roberts, et al., 2005; Bouchama, Kwaasi, et al., 2007; Bouchama et al., 2011, 2008; Dehbi et al., 2010; Roberts et al., 2008). This model enables direct organ and tissue examination after inducing heat stroke, addressing the limitations inherent in post‐mortem human studies (Chao et al., 1981; Malamud et al., 1946; Sohal et al., 1968). Human post‐mortem analyses, while informative, typically represent only the end‐stage of heat stroke, often overlooking crucial early and intermediate pathological changes. Furthermore, post‐death autolytic processes can alter tissue characteristics, leading to possible misinterpretations of disease progression (Nadol & Burgess, 1985).

Furthermore, the real‐time observations and analyses allowed by the baboon model capture the evolving pathophysiological changes of heat stroke. This approach is pivotal for monitoring acute responses such as the initial inflammatory cascade, coagulation abnormalities and early cellular stress responses, which are vital in the early stages of heat stroke but remain unobservable in post‐mortem human analyses. The physiological and anatomical similarities between baboons and humans, especially in the cardiovascular, neurological and thermoregulatory systems, make this model highly representative of human heat stroke (Phillips et al., 2014). Baboons' advanced cognitive and motor functions are particularly suitable for studying central nervous system (CNS) injuries in heat stroke, facilitating a more accurate understanding of both early and delayed CNS pathologies, which are prominent in this condition (Phillips et al., 2014). Additionally, the close similarity in genetic makeup of baboons and humans ensures more translatable insights into molecular and cellular responses to heat stress (Phillips et al., 2014).

Building upon this foundational understanding, our research focused on key pathogenic mechanisms, including inflammation, coagulation, cell death and stress response, and explored therapeutic strategies like anti‐inflammatory, anticoagulant, antiapoptotic and endothelial barrier function protection (Bouchama, Kwaasi, et al., 2008, 2007b; Bouchama et al., 2011). This narrative review synthesizes findings from these studies (Table 1) highlighting the significance of the baboon model in advancing our knowledge of heat stroke and developing innovative therapies.

**TABLE 1:** Summary of publications utilizing the baboon model of heat stroke.

Reference	Objective	Methods	Findings
Bouchama, Roberts, et al. (2005)	Investigate inflammatory, haemostatic and clinical changes in an experimental model for heat stroke	Anaesthetized baboons subjected to heat stress; measurement of inflammatory and haemostatic markers, liver and brain histopathology, and clinical outcomes	Severe heat stroke exhibited systemic inflammation, activated coagulation, liver and brain damage, and higher mortality than moderate heat stroke
Bouchama, Ollivier, et al. (2005)	Analyse the systemic inflammatory response of heat stroke	Anaesthetized heat‐stressed baboons assessed for systemic inflammatory response	Heat stroke elicited a complex systemic inflammatory response associated with the outcome
Bouchama, Kwaasi, et al. (2007)	Assess the efficacy of glucocorticoids in protecting against heat stroke	Anaesthetized heat‐stressed baboons treated with dexamethasone or control; evaluation of mortality, organ dysfunction and inflammatory markers	Dexamethasone did not protect against heat stroke and exacerbated tissue injury and organ dysfunction
Roberts et al. (2008)	Study of the pathogenic mechanisms of heat stroke	Anaesthetized baboons subjected to heat stress; assessment of tissue injury, cell death and inflammatory responses	Severe heat stroke resulted in widespread haemorrhage, thrombosis, leukocyte migration, microvascular endothelium injury and apoptosis
Bouchama et al. (2008)	Evaluate the effect of recombinant activated protein C on inflammation, coagulation, and cell and organ injury in heat stroke	Anaesthetized heat‐stressed baboons treated with recombinant activated protein C or control; measurement of endothelial injury, procoagulant microparticles and other markers	Recombinant activated protein C attenuated endothelial injury with no effect on organ injury and survival
Dehbi et al. (2010)	Investigate the potential prognostic value of Hsp‐72 in heat stroke	Anaesthetized heat‐stressed baboons were examined for Hsp‐72 expression; assessment of tissue damage and Hsp‐72 into circulation	Heat stroke led to differential induction of Hsp‐72 in various organs; extracellular release of Hsp‐72 was correlated with tissue damage
Bouchama et al. (2011)	Investigate whether tissue factor is the trigger for activation of coagulation in heat stroke.	Anaesthetized heat‐stressed baboons treated with recombinant nematode anticoagulant protein c2, inhibitor of tissue factor–factor VII or control; assessment of coagulation activation, fibrinolysis and inflammation	Tissue factor–factor VIIa initiates coagulation activation in heat stroke; coagulation inhibition did not affect fibrinolysis and inflammation, organ injury and survival

## 2 OVERVIEW OF THE BABOON MODEL OF HEAT STROKE

### 2.1 Clinical presentation

Classic heat stroke presents with a rapid elevation in core temperature, often to a temperature exceeding 40°C, accompanied by neurological changes and varying degrees of organ dysfunction following exposure to high ambient temperatures (Bouchama et al., 2022). It manifests in moderate or severe forms. Moderate heat stroke responds well to cooling and supportive therapy, while severe heat stroke results in profound, sustained multiorgan injury, often refractory to intensive care unit care, leading to high mortality (Argaud et al., 2007; Bouchama, 2006; Bouchama, Cafege, et al., 1991; Dematte et al., 1998; Misset et al., 2006). This categorization into moderate and severe forms is crucial for understanding the pathophysiological impact of heat stroke and guiding clinical management.

### 2.2 Development of the baboon model

Our baboon model of heat stroke was designed to emulate the clinical presentations and outcomes observed in human patients. We employed a detailed heat stress protocol (Supporting Information Table S1) in fully anaesthetized and intravenously hydrated baboons (*Papio hamadryas*), following approval from the Institutional Animal Care and Use Committee (IACUC) (Bouchama, Roberts, et al., 2005). This protocol induced heat stroke by exposing the baboons to environmental heat between 44 and 47°C and relative humidity of 37–39% within a modified neonatal incubator. Sham heated animals in the same neonatal incubator pre‐set at a temperature of 27.7 ± 0.5°C and humidity of 36 ± 3.1% for an equivalent time served as a control group.

Initially, our research focused on creating a severe model of classic heatstroke, mirroring human conditions where haemodynamic instability signals poor prognosis. Based on earlier studies (Austin & Berry, 1956; Malamud et al., 1946) and subsequent observations (Dematte et al., 1998; Hart et al., 1982), we identified a systolic arterial pressure (SAP) below 90 mmHg, which occurs in baboons at core temperatures exceeding 43°C as indicative of severe heatstroke. However, this model resulted in high mortality rates. To encompass the full spectrum of heatstroke severity, we introduced a moderate heatstroke category, characterized by a maximum rectal temperature of 42.5°C identified as the highest before the onset of any haemodynamic instability and hypotension (Bouchama, Roberts, et al., 2005). Following heat stress, the animals were promptly removed from the incubator upon reaching predetermined endpoints for severe or moderate heatstroke and passively cooled in a room maintained at 26−29°C. To prevent hypoglycaemia and maintain a mean arterial pressure (MAP) >60 mmHg, dextrose 5% and normal saline were administered, respectively. Severe and moderate heat stroke closely replicated the pathophysiological changes observed in humans during heat stroke (Supporting Information Table S2) (Bouchama, Ollivier, et al., 2005; Bouchama, Roberts, et al., 2005; Bouchama, Kwaasi, et al., 2007; Bouchama et al., 2011, 2008; Dehbi et al., 2010; Roberts et al., 2008).

### 2.3 Laboratory investigations

Our investigations involved collecting blood samples up to 3 h during passive cooling and monitoring for up to 72 h. This approach allows us to capture dynamic pathophysiological changes as they unfold before recovery or death. Tissue samples were taken immediately *post mortem* or at 72 h after euthanasia. Such temporal resolution is not achievable in human post‐mortem studies. Consequently, our methodology provides a more comprehensive understanding of the progression of heat stroke, particularly in the critical early stages, thereby enhancing our overall understanding of the disease with two distinct outcome phenotypes. Moreover, we employed various techniques such as light and electron microscopy histology, immunohistochemistry, confocal microscopy, terminal deoxynucleotidyl transferase dUTP nick end labelling (TUNEL) for apoptotic cell death, and DNA fragmentation electrophoresis immediately *post mortem* to minimize autolytic changes. These methods allowed us to examine structural and ultrastructural changes and describe the mechanisms underlying multiorgan injury and cell death caused by heat stroke.

### 2.4 Relevance to human conditions

In our study, both moderate and severe heat stroke required normal saline to maintain a mean arterial pressure (MAP) ≥60 mmHg. However, in severe heat stroke, despite greater fluid volume, MAP remained low due to a sustained decrease in diastolic arterial pressure, mirroring the distributive shock state seen in human heat stroke cases (Bouchama, Dehbi, et al., 2007), further validating the model's relevance to human conditions. This haemodynamic profile, largely attributed to excessive inflammatory responses and vascular endothelium dysfunction, including vascular leak and vasodilatation, was confirmed through the demonstration of exuberant systemic inflammatory response and endothelial cell damage observed by scanning electron microscopy (Roberts et al., 2008). Conversely, moderate heat stroke in baboons did not lead to shock but was characterized by inflammation, as indicated by increased interleukin (IL)‐6 and leukocytosis, and mild organ injury, especially in the pallidum and cerebellum (Bouchama, Roberts, et al., 2005). These changes, observable under light microscopy, manifested as lethargy and limb weakness during recovery, underscoring the brain's vulnerability even in moderate heat stroke scenarios.

### 2.5 The concept of critical thermal maximum

Critical thermal maximum (CTM), defining a temperature level and duration threshold beyond which near‐lethal or lethal injury occurs, is crucial in our baboon model for distinguishing between moderate and severe heat stroke (Bynum et al., 1978). We identify moderate heat stroke at a threshold of 42.5°C and severe heat stroke at >43°C, both over a duration of approximately 3 h, consistent with CTM parameters (Supporting Information Table S1). The 42.5°C threshold indicates significant yet manageable physiological stress, aligning with observations in anaesthetized humans where a CTM of 41.6−42.0°C over 1 h is linked to mild health outcomes (Bynum et al., 1978). Conversely, severe heat stroke surpasses the CTM, leading to rapid deterioration and heightened mortality risk. Our study thus not only categorizes heat stroke severity but also enhances understanding of physiological limits to extreme heat, increasingly relevant due to global temperature trends.

## 3 PATHOGENIC MECHANISMS OF ORGAN INJURY

### 3.1 Multiorgan injury

**FIGURE 1:**
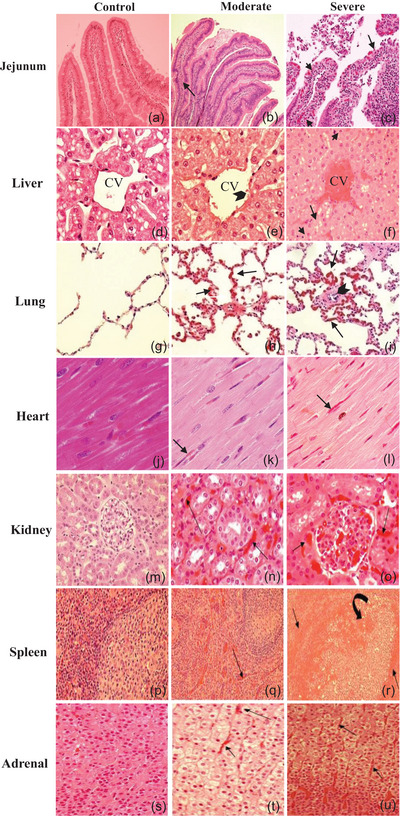
Microvascular alterations across organs in heat stroke. (a–c) Jejunum. (a) Normal intestinal villi in a control animal. (b) Moderate heat stroke shows limited villous capillary distension (arrow). (c) Severe heat stroke exhibits pronounced jejunal villous capillary swelling, red blood cell stasis (arrows), and amplified inflammatory cell presence with loss of surface epithelium. (d–f) Liver. (d) Control animal liver is unremarkable. (e) Moderate heat stroke shows sporadic platelets adhered to sinusoidal endothelium (arrowhead). (f) Extensive pan‐lobular sinusoidal hepatic congestion in severe heat stroke, spanning portal triads to central veins (CV), with amassing of leukocytes in sinusoids (arrows). (g–i) Lung. (g) Control animal lung is unremarkable. (h) Mild to moderate capillary congestion and leucocytes infiltration in moderate heat stroke (arrows). (i) Pronounced alveolar capillary distension with red blood cell engorgement (arrows), increased inflammatory cells, and partial arteriolar occlusion by thrombus (arrowhead) in severe heat stroke. (j–l) Heart. (j) Normal myocardial capillary in control. (k, l) Myocardial capillary engorgement (arrows) in both moderate (k) and severe (l) heat stroke. (m–o) Kidney. (m) Normal peritubular and glomerular capillaries in control. (n, o) Pronounced amplification and red blood cell accumulation of peritubular capillaries in moderate (arrows (n)) and severe (arrows (o)) heat stroke. Glomerular capillaries are engorged in severe heat stroke with expanded mesangial cellularity in both moderate (arrows (n)) and severe (arrows (o)) heat stroke. (p–r) Spleen. (p) Normal spleen architecture in control. (q) Moderate red pulp engorgement in moderate heat stroke (arrow). (r) Extensive red pulp congestion in severe heat stroke (straight arrows) with extension into the white pulp (curved arrow). (s–u) Adrenal. (s) Normal adrenal gland in control. (t, u) Pronounced sinusoidal capillary swelling and engorgement in the zona fasciculata in both moderate (arrows (t)) and severe (arrows (u)) heat stroke. Original illustrations: a–c, f, j–l, m–o, q–u. Adapted with permission from Bouchama et al. (2022): d, e. Adapted with permission from Roberts et al. (2008): g–i, p.

Heat stroke leads to early multiorgan injuries, increasing morbidity and mortality (Yezli et al., 2023). The baboon model revealed vascular dysfunction, inflammation, thrombosis, haemorrhage, organ disruption and cell death mechanisms in multiple organs (Figures 1 and 2) (Bouchama, Roberts, et al., 2005, Bouchama et al., 2011; Roberts et al., 2008). Likewise, structural and ultrastructural evidence of organ disruption was evident in the jejunum, lung, liver, myocardium and kidneys (Figure 2). Further, cellular organelles were disrupted, such as lysosomes losing their internal structures or mitochondrial cristae assuming a ring shape (Figure 2m–t).

FIGURE 2Organ structural and ultrastructural disruptions in heat stroke. (a) Extensive desquamation of jejunal surface epithelium, with desquamated fragments in lumen (arrows). (b) Increased thickening of pulmonary alveolar walls, with increased cellular content (arrows). (c) Disruption of liver parenchymal architecture, with separation of hepatocytes by haemorrhage (arrows). (d) Minimal myocardial changes in control heat stroke. (e, f) Myocardial oedema (arrowheads) and fibre fragmentation (arrows) in moderate (e) and severe (f) heat stroke. (g) Normal renal tubules. (h) Minimal tubular changes in moderate heat stroke. (i) Tubular granular casts (continuous arrow), epithelial debris (arrowhead), and red blood cell casts (dashed arrow) in severe heat stroke. (j) Normal glomeruli. (k, l) Increased glomerular mesangial cellularity in both moderate and severe heat stroke. (k) Narrowed glomerular capillaries in moderate heat stroke. (l) Dilated glomerular capillaries in severe heat stroke with amplified intra‐glomerular platelets (arrow) and extravasation of red blood cells into Bowman's space (dashed arrow (l)). (m) Normal microvilli in control. (n) Limited microvillus widening (arrow) in moderate heat stroke. (o) Disorganized jejunal microvilli with terminal bulbous distensions (arrows) in severe heat stroke. (p–r) Liver and kidney ultrastructure. (p) Control animal hepatic microvilli are normal (continuous arrows), projecting into the space. (q, r) The space of Disse is obliterated in both moderate (q) and severe (r) heat stroke, with reduction of hepatic microvilli to irregular stubs (arrows (q)). Lysosomes lose their internal structure (continuous arrow (r)), and mitochondrial cristae assume a ring profile (dashed arrows (r)). (Original magnification ×15000.) (s, t) Cytoplasmic haemosiderin accumulation in renal tubular epithelium in moderate heat stroke (arrow (s)) and activated platelets (arrow (t)) interacting with red blood cells in glomerular capillary in severe heat stroke. (Original magnification ×2950.) Original illustrations: b, c, d–f, m, s. Adapted with permission from Bouchama et al. (2022): a, g–l, p–r. Adapted with permission from Roberts et al. (2008): n, o, t.

**Figure:**
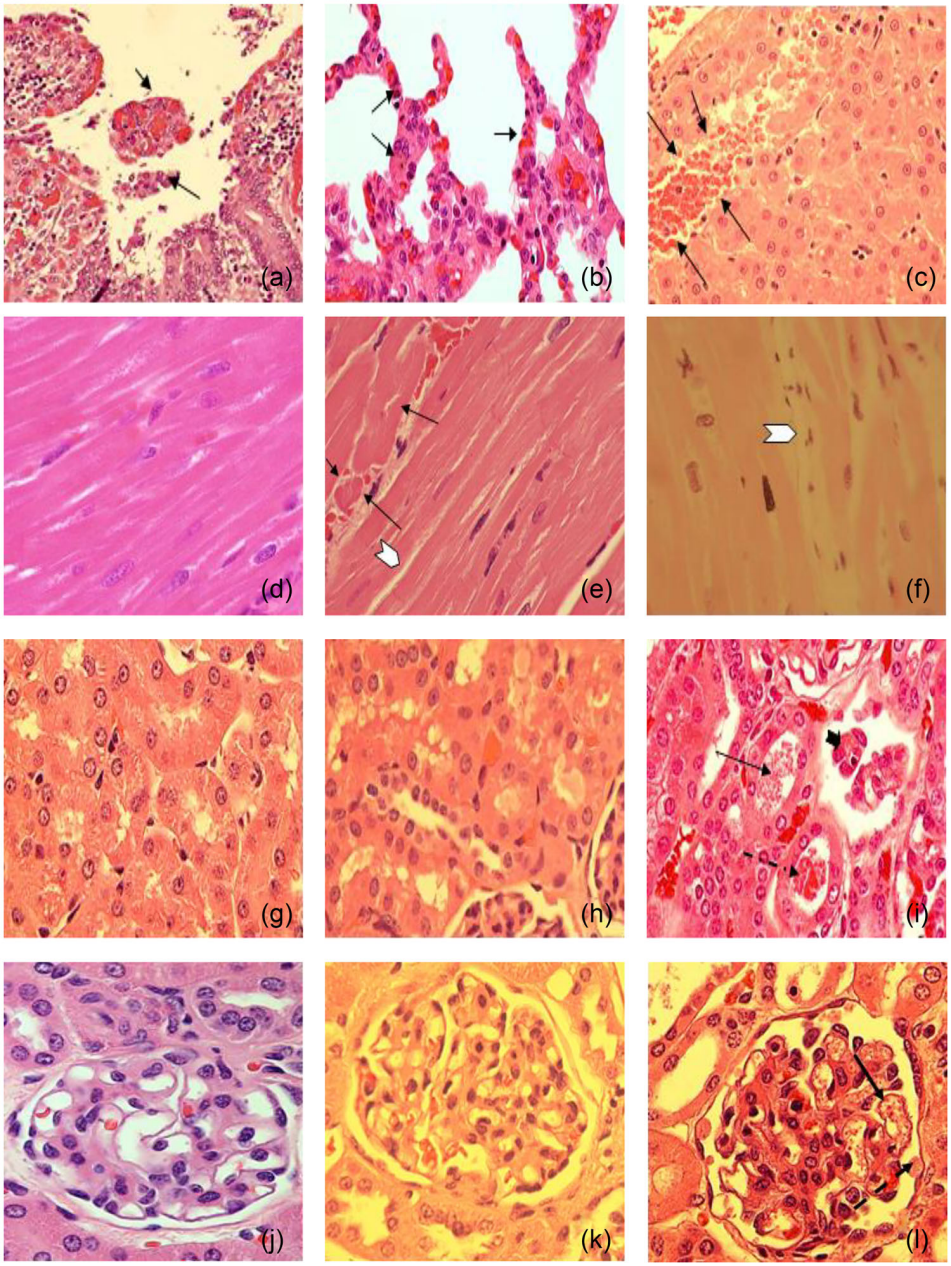

**Figure:**
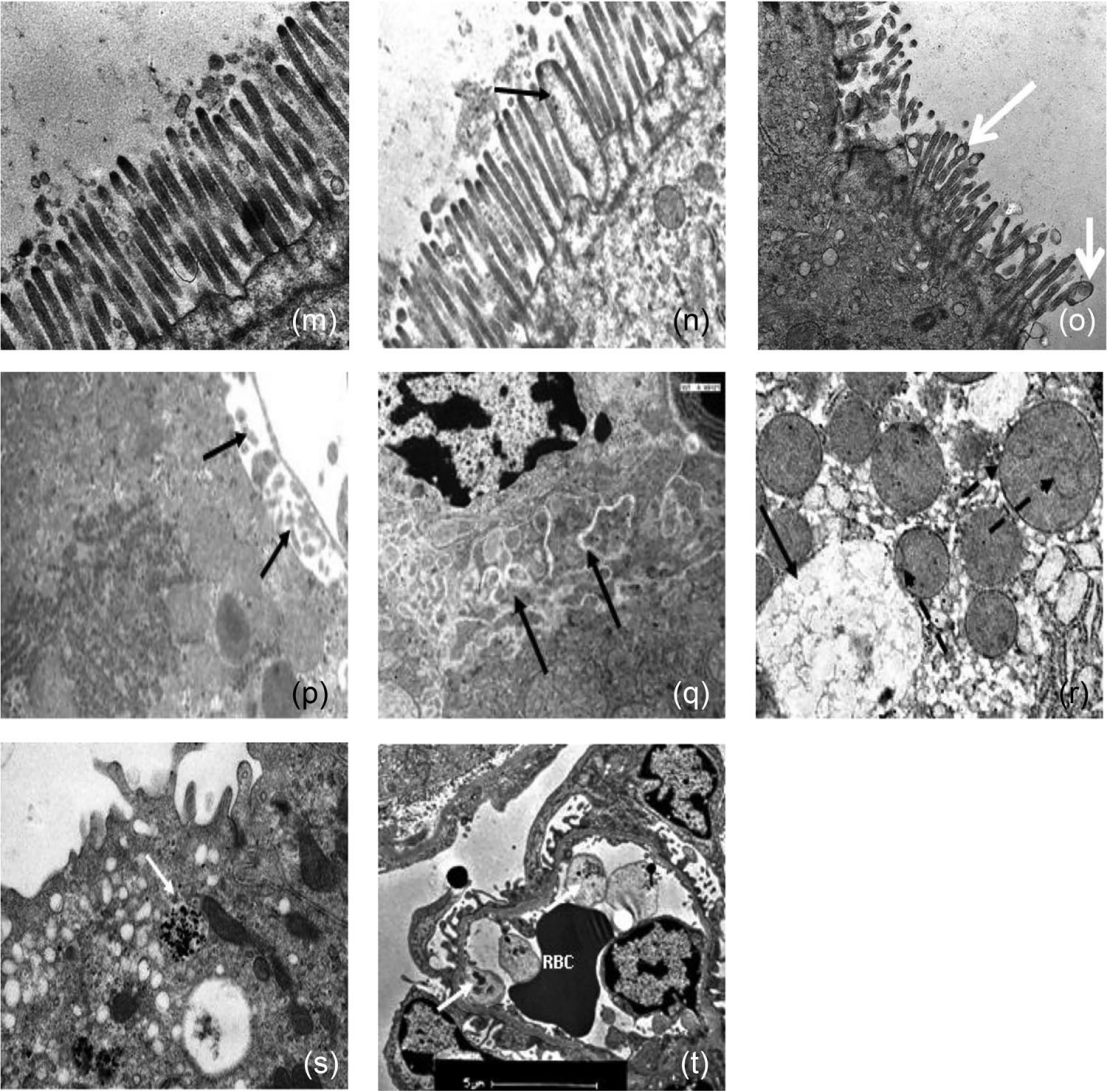

### 3.2 Endothelial injury

As shown in Figure 3, endothelial injury manifested with distinctive features, including cytoplasmic ballooning, myelin whorl formation, widening of intercellular gaps leading to endothelial barrier breaches, and apoptosis of endothelial cells. Additionally, increased Weibel–Palade body expression and erythrocyte membrane deformities including fragmentation and fissuring indicated functional impairment (Figure 3A, B). It is noteworthy that Weibel–Palade bodies are responsible for synthesizing and releasing von Willebrand factor (vWF) antigen and P‐selectin, which play pivotal roles in haemostasis and inflammation, respectively (Nightingale et al., 2018). Immunohistochemistry further revealed increased expression of vWF antigen in many organs, indicative of systemic endothelial cell activation (Figure 3C). This observation is supported by elevated plasma levels of recognized biomarkers of endothelial cell activation/injury in baboons (Bouchama, Roberts, et al., 2005; Bouchama et al., 2008) and humans (Bouchama, Hammami, et al., 1996; Shieh et al., 1995). Overall, these findings underscore the role of vascular injury and dysfunction as central components of heat stroke pathophysiology.

FIGURE 3Endothelial injury, leukocyte infiltration, and thrombus formation in heat stroke. Panel (A). (a) Ultrastructural capillary congestion with luminal occlusion by continuous red blood cell–platelets–fibrin thrombus (R, P and F) and spillage of platelets through a break in the vessel wall (arrow). (Original magnification ×2900.) (b) Transcapillary extravasation of red blood cells (continuous arrow), platelets (dashed arrow) and leukocytes (arrowhead) interacting with subendothelial collagen fibres (asterisk). Transmission electron microscopy. (Original magnification ×5200.) (c, d) Ultrastructural changes in endothelial cells include increased villi formation (arrowheads (c, d)) and myelin whorls (arrow (c)) in moderate heat stroke. (Original magnification ×5200.) Increased expression of Weibl–Palade bodies (arrows (d)) in moderate heat stroke. (Original magnification ×8900.) (e) Attenuation of capillary endothelium cytoplasm (white curved arrow) with neutrophil (Neut) that passed from lumen into interstitial tissue, interacting with collagen fibres (*). (f) Cytoplasmic bleb formation in venular endothelial cell (arrow) with intra‐luminal erythrocyte. (Original magnification ×3900.) Panel (B). (a–c) Haematological changes in heatstroke are seen from T+0 h (onset of heatstroke) to T+3 h. (a) Red blood cells are unremarkable in control animal (Wright–Geimsa stain). (b, c) Red blood cell fragmentation in severe HS animals at T+0 (continuous arrows (b)) and T+3 (arrows (c)) with microspherocytes (dashed arrow (c)). Lymphocyte apoptosis, consisting of nuclear condensation and fragmentation, is present in moderate and severe heatstroke (arrowhead (b)). (d–f) Scanning electron micrographs of red blood cells display normal red blood cell in control (d), papular eruptions (arrow (e)) appear two hours from the onset of heat stroke, progressively getting more numerous at three hours associated with membrane fissuring (arrow (f)) and adherent particulate material (dashed arrows (f)). Panel (C). Expression of vWF in pulmonary (a–c), myocardial (d–f), hepatic (g–i), and glomerular (j–l) vascular beds. It is more marked in severe heat stroke (c, f, i, l) than moderate heat stroke (e, h, k), except in the lungs where the greatest increase is in moderate heat stroke (b). Original illustrations: panel (A): c, d; panel (B): a–f. Adapted with permission from Bouchama et al. (2022): panel (A): b. Adapted with permission from Roberts et al. (2008): panel (A): a, e, f; panel (C): a–l.

**Figure:**
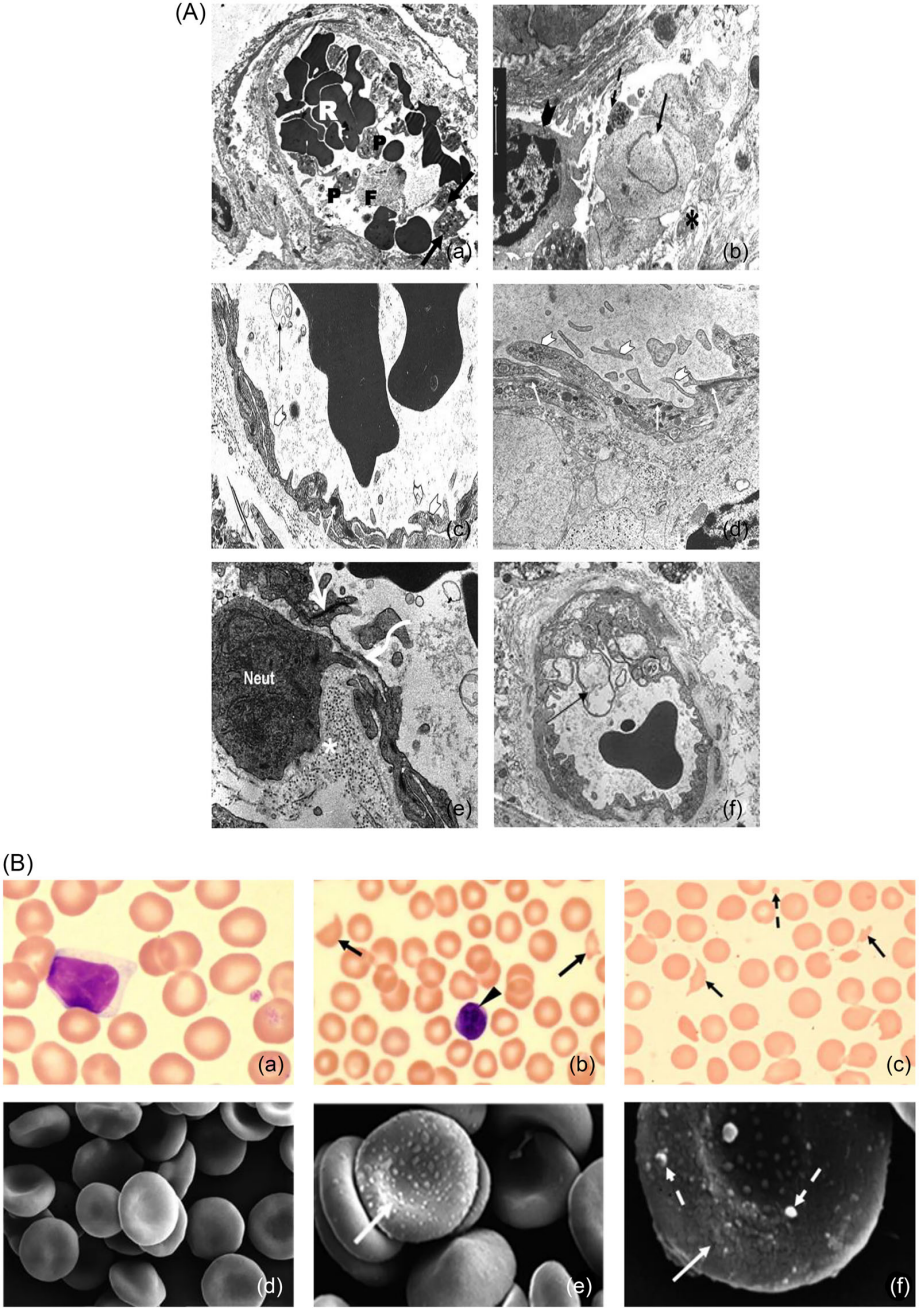

**Figure:**
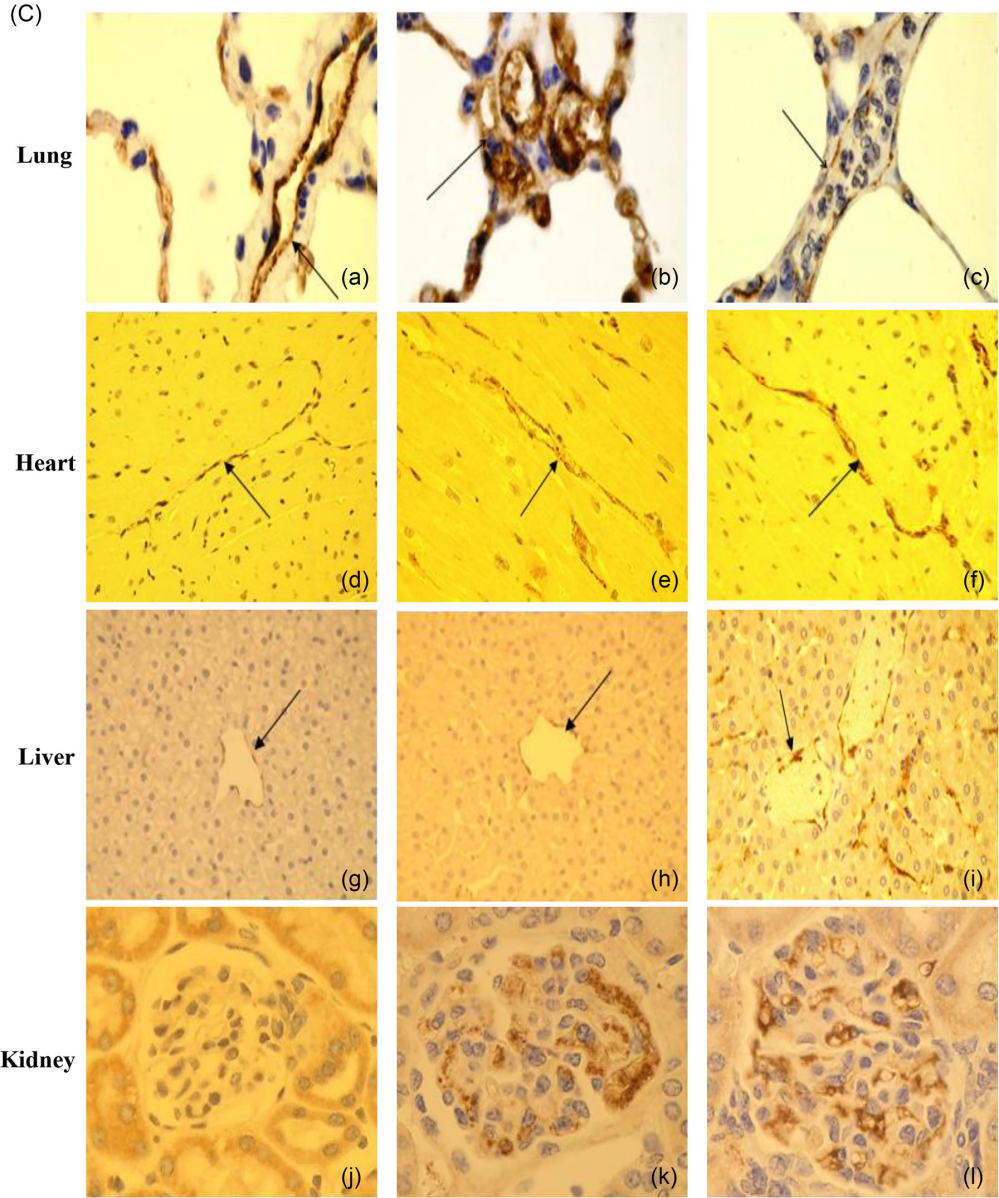

### 3.3 CNS injury

CNS injury in heat stroke often resembles a stroke, with symptoms like delirium, unconsciousness and occasional post‐cooling seizures (Yaqub & Al Deeb, 1998; Yaqub et al., 1986). Brain computed tomography and magnetic resonance imaging rarely reveals structural damage explaining these symptoms, leaving acute brain injury mechanisms largely unknown (Bouchama et al., 2022). Long‐term studies found up to one‐third of survivors to be suffering cerebellar syndrome and cognitive dysfunction (Argaud et al., 2007; Bouchama et al., 2022; Dematte et al., 1998). Brain imaging typically shows cerebellar atrophy, with potential hippocampus and thalamus damage (Bouchama et al., 2022).

**FIGURE 4:**
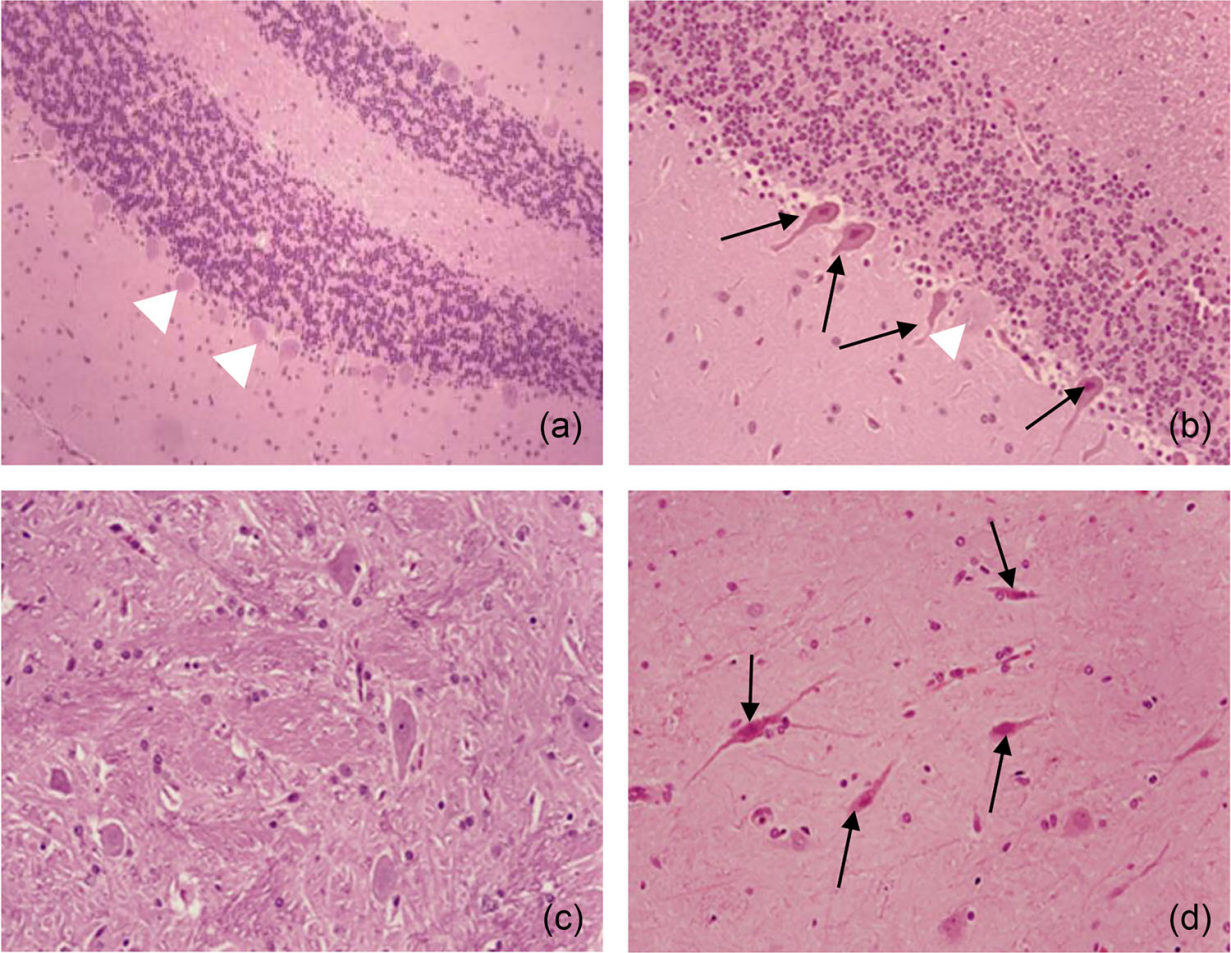
Cerebral histopathology in heat stroke. Haematoxylin and eosin staining of brain tissue sections in control and heat stroke study groups. (a) Normal cerebellum with normal Purkinje cells in sham‐heated controls (white arrowheads). (b) Early Purkinje cell necrosis (continuous arrows) and normal Purkinje cells (white arrowheads) in moderate heat stroke. (c) Normal pallidum in sham‐heated controls. (d) Widespread neuronal necrosis in severe heat stroke (arrows). Adapted with permission from Bouchama, Roberts, et al. (2005).

Autopsy studies in patients with persistent neurological damage revealed severe loss of Purkinje cells, with evidence of neuron apoptosis in the thalamus (Bazille et al., 2005). These lesions differ from anoxia and ischaemia‐related neuronal changes, suggesting different injury mechanisms in heat stroke (Bazille et al., 2005). Replicating brain injury, particularly the loss of Purkinje cells and thalamic neuronal death observed in humans, is crucial for developing brain preservation strategies. Baboon brain histopathology during heat stroke mirrored human necropsy studies, showing cytoplasmic eosinophilia and nuclear pyknosis in scattered neurons of the hippocampus, pallidum and cerebellar Purkinje cells (Figure 4) (Bouchama, Roberts, et al., 2005). These findings underline the significance of understanding the mechanisms at play, as the brain regions affected by heat stroke are pivotal for memory, motor control, and coordination.

## 4 CELL DEATH MECHANISMS

### 4.1 Cell death

Research conducted using cell lines and animal models has provided compelling evidence that heat exposure can initiate both necrotic and apoptotic cell death pathways (Sakaguchi et al., 1995). Notably, in rats subjected to moderate whole‐body hyperthermia, there was a consistent pattern of early and sustained apoptotic cell death observed in organs such as the jejunum, spleen, bone marrow and thymus (Sakaguchi et al., 1995). Collectively, these findings suggest that heat‐induced cell death may indeed play a significant pathogenic role in hyperthermic conditions, such as heat stroke.

The baboon model of heat stroke has confirmed the presence of necrosis and apoptosis as the predominant forms of cellular death (Figure 5). Apoptotic cell death was prominently observed in various organs, as evidenced by the TUNEL assay and DNA fragmentation analysis (Figure 5A). Immunostaining for caspase 3, a key protease involved in programmed cell death, showed increased expression in the spleen, lung and jejunum, co‐localizing with apoptotic cells (Figure 5B). These findings provide strong evidence of active and widespread apoptotic cell death in heat stroke.

FIGURE 5Evidence of necrotic and apoptotic cell death in moderate and severe heat stroke. Panel (A). (a) Small intestinal lamina propria showing apoptotic cell death (dashed white arrow), autophagic vacuoles (continuous arrow) and necrotic cell death (N) (×1650 magnification). (b) Apoptosis in jejunal capillary endothelial consisting of marked condensation of nuclear heterochromatin (arrow) (original magnification ×8900). (c) Apoptotic changes consisting of nuclear condensation and cytoplasmic fenestration in alveolar capillary neutrophil (Nt) and lymphocyte (Ly) (original magnification ×3900) in severe heatstroke. (d–r) Fluorescence labelling of apoptotic nuclei by terminal deoxynucleotidyl transferase biotin‐dUTP nick end labelling (TUNEL) assay. (d–f) Jejunum; (g–i) liver; (j–l) lung; (m–o) heart; (p–r) kidney. Apoptosis shown by bright fluorescent nuclear or cytoplasmic staining. Control animal (d, g, j, m, p) shows minimal apoptosis (arrows (d, j)). Mild to moderate bright nuclear and cytoplasmic staining are seen in moderate heat stroke (e, k, n) and focally in the kidney (q). Marked changes are observed in jejunum in (f), intra‐hepatic leukocytes (h, i) and lungs (l), myocardium (o), and renal tubules (r) in severe heat stroke. Panel (B). (a) Fluorescence labelling of apoptotic nuclei by TUNEL assay. Minimal TUNEL‐positive cells (bright green) are present in spleen of control baboons. (b) Numerous TUNEL‐positive cells are observed in severe heat stroke. (c, d) Immunohistochemistry for active caspase‐3 in spleen from severe heat stroke and control baboons. (c) No positive staining is seen in control spleen. (d) Positive brown staining for active caspase‐3 is present in spleen from severe heat stroke. (e) Semi‐quantitative analysis of TUNEL‐positive cells per field of view in severe heat stroke and 48 h after moderate heat stroke compared to control. Apoptotic cells expressed as fold‐increase from control. (f) DNA fragmentation in splenic tissue is increased in moderate (MHS) and severe heat stroke (SHS) compared to control (CHS) on gel electrophoresis. Original illustrations: panel (A): a, g–i, m–o; panel (B): e, f. Adapted with permission from Roberts et al. (2008): panel (A) b–f, j–l; panel (B): a–d.

**Figure:**
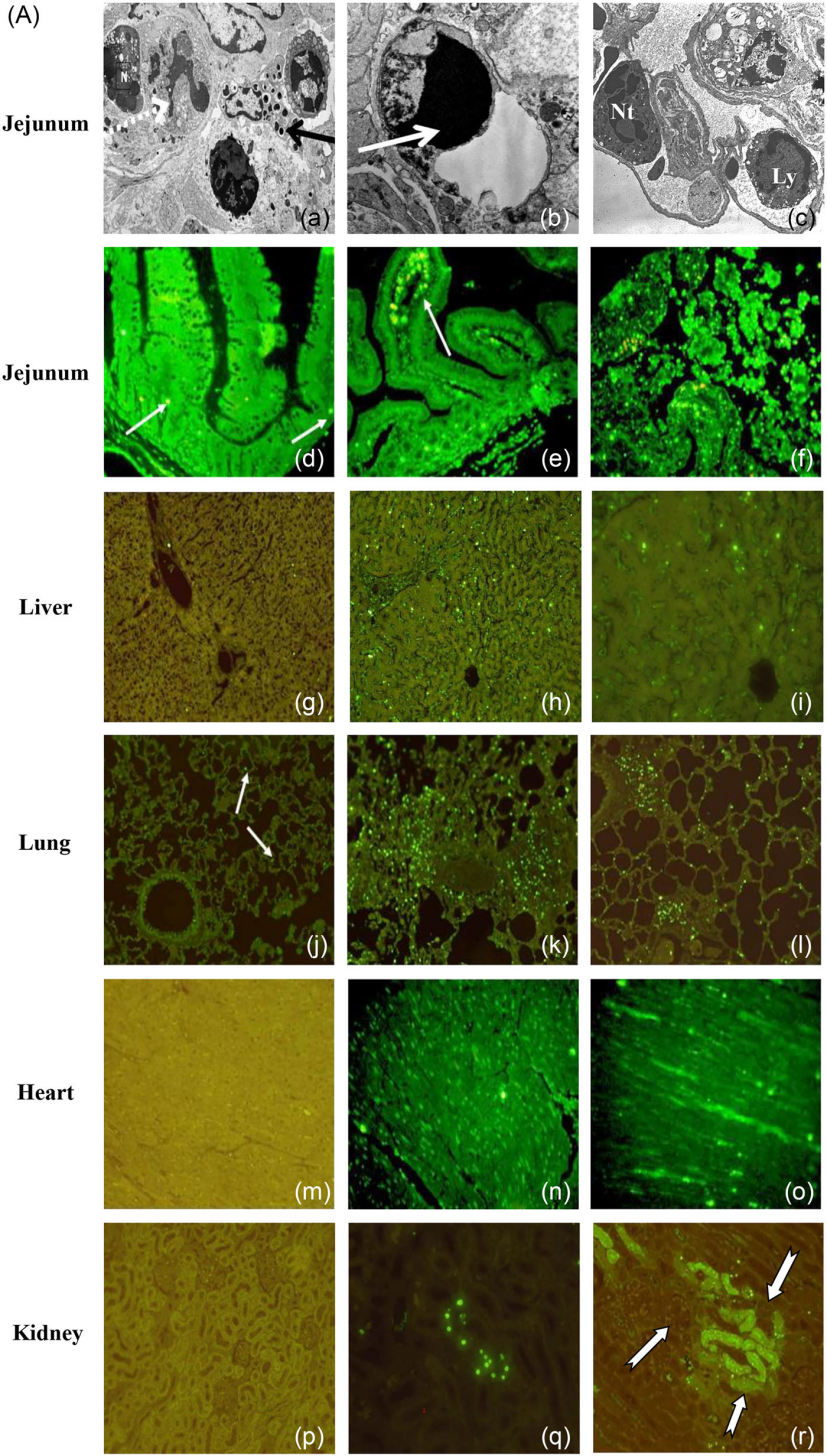

**Figure:**
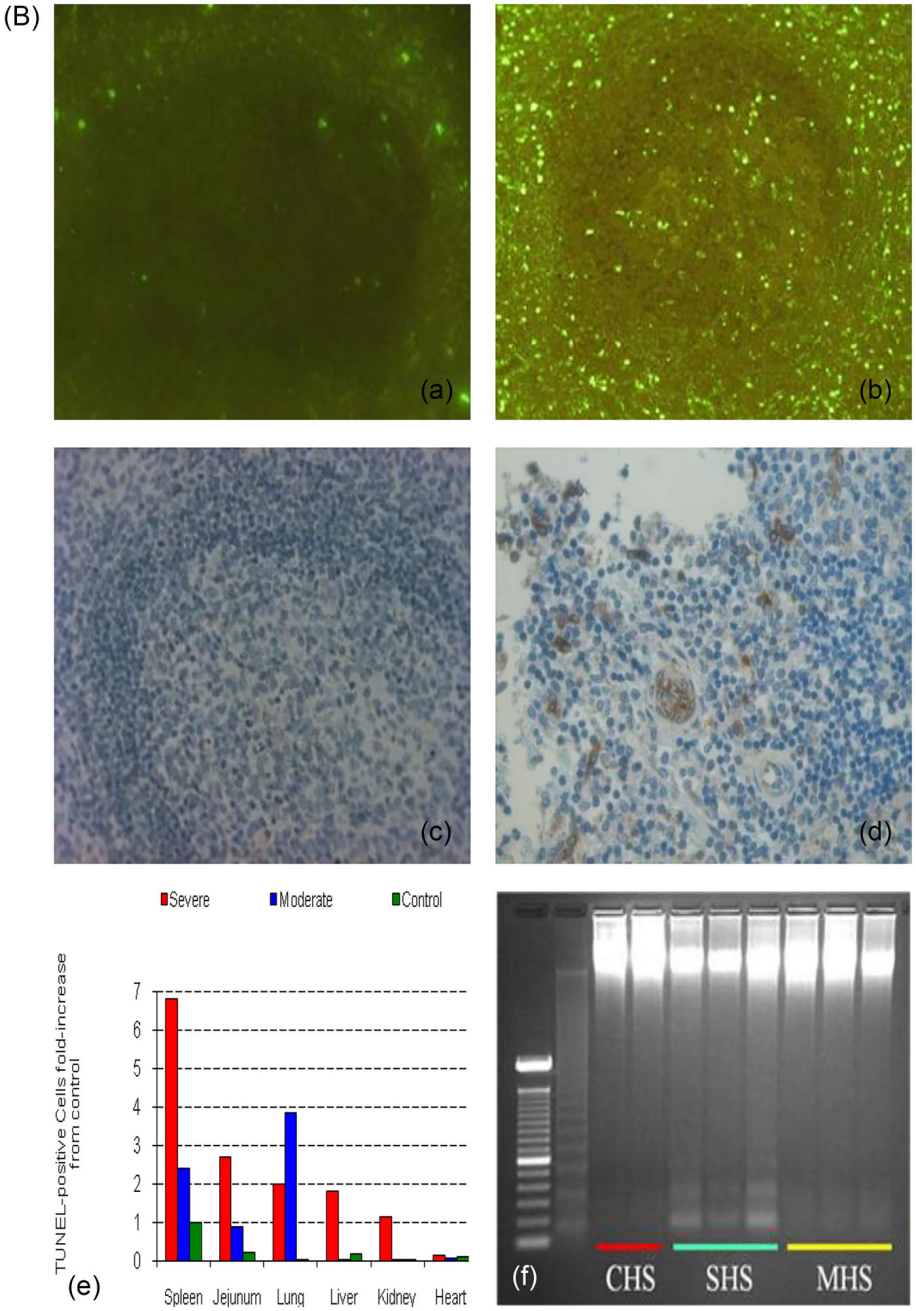

### 4.2 Apoptotic cell death

Apoptosis was observed in different cell types, including enterocytes, inflammatory cells, endothelial and splenic cells, myocardial myocytes, liver, and lung cells, displaying nuclear pyknosis, cytoplasmic swelling and fragmentation characteristic of apoptotic cell death (Figure 5A) (Roberts et al., 2008; Taatjes et al., 2008). Intriguingly, our baboon study unveiled a distinct organ‐specific susceptibility to heat‐induced apoptotic cell death (Figure 5B). The spleen, gut and lungs showed higher levels of apoptosis compared to organs like the liver, heart and kidneys (Roberts et al., 2008). This organ‐specific pattern of cell death emphasizes the need for further research aimed at unravelling the distinctive responses of each organ. This research should pave the way for the development of tailored therapeutic interventions that precisely address the unique requirements and vulnerabilities of affected organs.

## 5 IMMUNE RESPONSE IN HEAT STROKE

### 5.1 Systemic inflammation

Heat stroke triggers a complex inflammatory cascade characterized by the release of pro‐inflammatory cytokines, chemokines and anti‐inflammatory mediators (Bouchama et al., 1993; Leon & Helwig, 2010). Previous studies in rodent models and clinical observations have demonstrated elevated plasma levels of various cytokines in heat stroke, including pro‐inflammatory cytokines such as IL‐6, tumour necrosis factor‐αa (TNF‐α) and IL‐1β, and anti‐inflammatory cytokines such as IL‐10 (Bouchama, Parhar, et al., 1991; Bouchama et al., 2000, 1993; Leon & Helwig, 2010; Lin et al., 1997; Liu et al., 2000). These circulating cytokines reflect the systemic inflammation associated with heat stroke and suggest their potential contribution to organ injury (Bouchama, Parhar, et al., 1991; Bouchama et al., 1993; 2000; Leon & Helwig, 2010; Lin et al., 1997; Liu et al., 2000).

**FIGURE 6:**
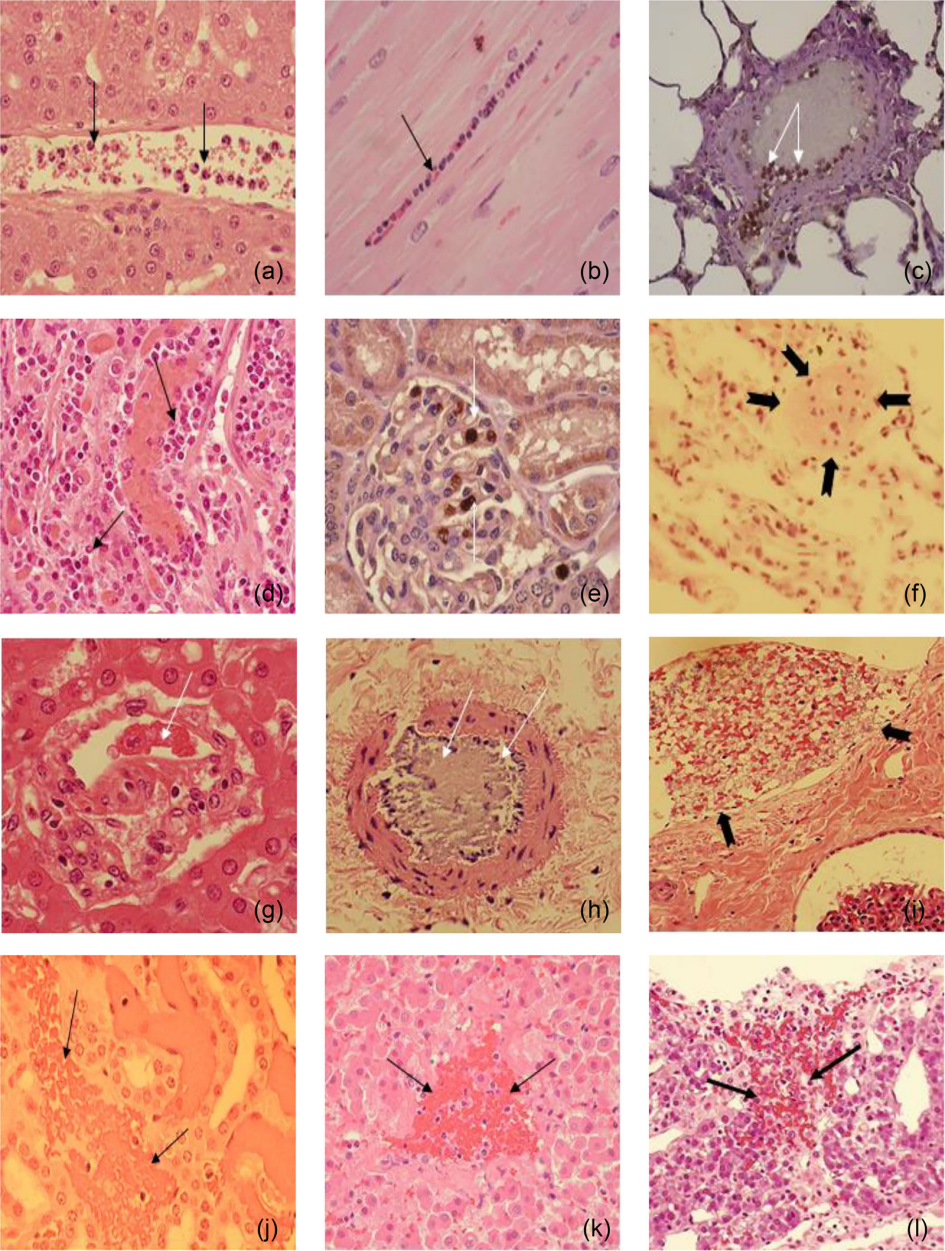
Histological evidence of inflammation and coagulation in heat stroke. (a–c) Neutrophil margination in hepatic sinusoids (with platelet aggregates) in moderate heat stroke (arrows (a)), myocardial capillary (arrow (b)) and pulmonary arteriole in severe heat stroke (arrows (c)). (d–f) Capillary dilatation, neutrophilic neutrophil margination and infiltration, and apoptosis in jejunal lamina propria (arrows (d)), intra‐glomerular neutrophils and focal pneumonic collection (arrows (e, f)) in severe heat stroke (MPO stain). (g–i) Thrombus deposition in portal tract (arrow (g)), pulmonary arteriole (arrows (h)) and renal vein tributary (arrows (i)) in severe heat stroke animals. (j–l) Intra‐parenchymal haemorrhage in kidney (arrows (j)), liver (arrows (k)) and adrenal (arrows (l)), in severe heat stroke. Original illustrations: c–e, i, j. Adapted with permission from Roberts et al. (2008): a, b, f–h, k, l.

The findings in baboons successfully mirror the cytokine response pattern observed in heat stroke patients. Furthermore, they contribute to a more comprehensive characterization of the temporal dynamics of this response, spanning from the onset of heat stroke to either recovery or fatal outcomes – an achievement that cannot be replicated in humans with heat stroke (Bouchama, Ollivier, et al., 2005; Bouchama, Roberts, et al., 2005). The research reveals that heat stroke triggers an early and complex systemic inflammatory response, marked by the simultaneous release (rather than a sequential release) of pro‐inflammatory cytokines such as IL‐6 and IL‐8, and anti‐inflammatory cytokines like IL‐10. Notably, the levels of these cytokines are proportionate to the severity of heat stroke (Bouchama, Ollivier, et al., 2005). The study also shows increased soluble tumour necrosis factor receptor I and II and IL‐1 receptor antagonist (sTNFrI and II, IL‐1‐ra), and a significant decrease in regulatory cytokine (IL‐12p40) in moderate and severe heat stroke (Bouchama, Ollivier, et al., 2005). Histopathological examination provided evidence of inflammation, and ultrastructural analysis reveals leukocyte infiltration into multiple organs (Figures 3A and 6) (Roberts et al., 2008). Neutrophils are observed in various organs, supporting the involvement of systemic and multiorgan tissue inflammation in heat stroke pathophysiology.

### 5.2 Danger signals

Endotoxin leakage from heat‐induced gut damage (Bouchama, Parhar, et al., 1991; Gathiram et al., 1988; Leon & Helwig, 2010) and the release of danger signals or alarmins, such as HMGB1 (Dehbi et al., 2012) and HSP‐72 (Dehbi et al., 2010; Huisse et al., 2008), from necrotic cells have been implicated in initiating and propagating the inflammatory response in heat stroke. Studies in experimental rodent models have shown that neutralizing endotoxin or HMGB1 can prevent organ damage and improve survival (Dehbi et al., 2012; Gathiram, Wells, et al., 1987). Inhibition of the inflammatory response with corticosteroids (Liu et al., 2000), recombinant activated protein C (Chen et al., 2006) or IL‐1 receptor antagonist (Lin et al., 1997; Liu et al., 2000) has also been shown to prevent tissue injury and death, suggesting the potentially harmful effects of systemic inflammation in heat stroke.

However, these benefits could not be replicated in the baboon model as anti‐inflammatory agents, such as dexamethasone, exacerbated organ injury (Bouchama, Kwaasi, et al., 2007). These observations underscore the complexities of the inflammatory response in heat stroke and caution against the indiscriminate use of anti‐inflammatory agents in its treatment. More research is needed to unravel the complex mechanisms underlying the inflammatory response and identify appropriate therapeutic strategies to modulate it in heat stroke effectively.

## 6 COAGULATION ABNORMALITIES

### 6.1 Coagulopathy

In fatal heat stroke cases, widespread organ haemorrhage and necrosis are common, pointing to coagulation abnormalities as a key factor in organ injury (al‐Mashhadani et al., 1994; Chao et al., 1981; Malamud et al., 1946). Studies indicate that around 18% of heat stroke patients experience fatal bleeding, often associated with low platelet counts and decreased anticoagulant protein C levels, implying coagulation factor depletion (al‐Mashhadani et al., 1994; Bouchama, Bridey, et al., 1996). Post‐mortem studies have confirmed haemorrhage and necrosis in various organs (Chao et al., 1981; Malamud et al., 1946; Sohal et al., 1968).

Sensitive biomarkers have revealed initial coagulation changes in heat stroke patients (Bouchama, Bridey, et al., 1996). Coagulation and fibrinolysis activation occur rapidly, with increased thrombin and plasmin levels. Although cooling therapy normalizes core temperature, it reduces fibrinolysis due to release of plasminogen activator inhibitor (PAI)‐1, maintaining active coagulation and resulting in hypercoagulation (Bouchama, Bridey, et al., 1996). Reduced protein C and antithrombin levels indicate consumption, contributing to the prothrombotic environment. Rat studies show heat stroke induces disseminated intravascular coagulation (DIC), and administration of recombinant human‐activated protein C reduces tissue injury and improves survival (Chen et al., 2006). These findings strongly suggest that coagulopathy could serve as a pathogenic mechanism contributing to multiorgan injury, dysfunction and ultimately lethality in heat stroke. However, it remained unclear what triggers the activation of the coagulation cascade in this context. To address this question, we initially examined whether heat stroke in baboons mirrored the coagulation abnormalities observed in humans, including DIC.

**FIGURE 7:**
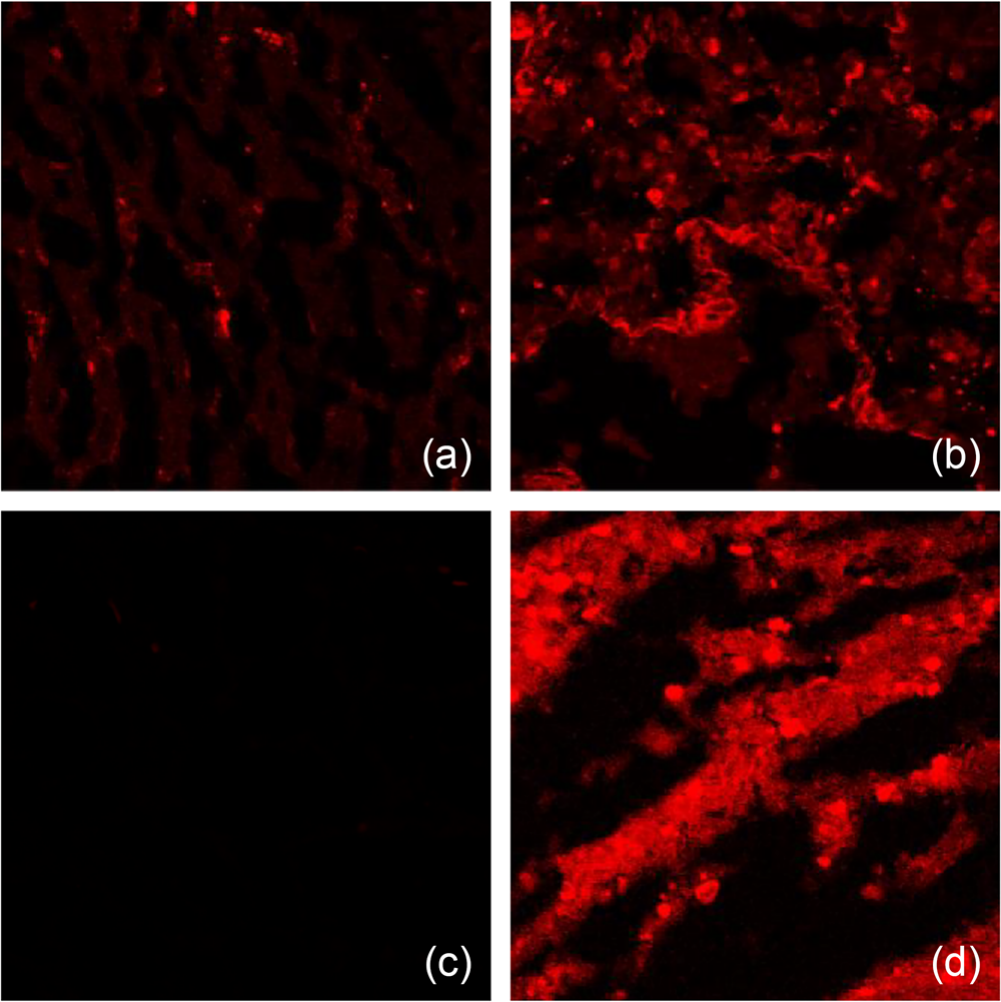
Tissue factor expression in visceral organs during heat stroke. No positive red staining for TF is visualized with confocal microscopy in jejunum (a) and spleen (c) from control as compared with positive staining for TF (red) jejunum (b) and spleen (d) in severe heat stroke. Adapted with permission from Roberts et al. (2008).

The heat‐stressed baboons indeed exhibited a coagulation‐fibrinolysis imbalance similar to what is seen in human heat stroke, resulting in a prothrombotic environment culminating in DIC in severe heat stroke (Bouchama et al., 2011, 2008; Roberts et al., 2008). Histopathological studies further confirmed thrombi formation in multiple organs, consistent with post‐mortem human findings (Figure 6) (Chao et al., 1981; Malamud et al., 1946; Roberts et al., 2008; Sohal et al., 1968). Additionally, elevated tissue factor (TF) expression was observed in the splenic and jejunal tissues of severe heat stroke baboons concomitantly with TF‐active microparticles release (Figure 7) (Bouchama et al., 2008; Roberts et al., 2008).

### 6.2 Role of tissue factor

Tissue factor (TF) is a cell surface protein that initiates the extrinsic coagulation pathway when exposed to the bloodstream (Pawlinski et al., 2004). It triggers a series of enzymatic reactions leading to thrombin generation and fibrin formation. In acute conditions like sepsis, activation of TF–FVIIa complex‐induced coagulation is implicated in multiorgan system dysfunction and death (Creasey et al., 1993).

To elucidate its role in heat stroke, we examined whether the TF–FVIIa complex served as the trigger for the observed coagulopathy in baboons (Bouchama et al., 2011). Our findings revealed that blocking the TF–FVIIa complex with recombinant nematode anticoagulant protein c2 (rNAPc2), a specific inhibitor, effectively prevented coagulation activation (Bouchama et al., 2011). This discovery firmly established the central role of the TF–FVIIa complex as the primary initiator of coagulation activation during heat stroke. Targeting this complex emerges as a promising therapeutic strategy to mitigate coagulation abnormalities and organ injury in heat stroke.

## 7 RESPONSE PATTERNS OF HEAT SHOCK PROTEINS

### 7.1 Intracellular heat shock proteins response

The heat stress response (HSR) is a cellular defence mechanism evolved to protect against heat‐induced toxicity (Richter et al., 2010). Heat shock proteins (HSPs), known as chaperones, are typically expressed at low levels in cells but become more abundant during heat stress. HSPs play a crucial role in identifying and correcting misfolded or aggregated proteins or directing them for degradation (Richter et al., 2010). Transcriptome analysis of laboratory rodents (Stallings et al., 2014) and humans (Bouchama et al., 2023) with heat stroke has revealed a significant upregulation of genes encoding HSP, co‐chaperones and chaperonins, indicating the robust activation of the HSR during heat stroke.

In laboratory rats, heat stress leads to the upregulation of HSP‐72 in various organs, with the extent of expression correlating with the severity of heat stress (Flanagan et al., 1995). Pre‐induction of HSP‐72 before heat stress has reduced organ damage and improved survival (Blake et al., 1990; Flanagan et al., 1995; Yang & Lin, 1999).

In our investigation of baboons with heat stroke, we observed up‐regulation of Hsp‐72 in specific organs, with the liver showing the highest expression, followed by the jejunum, heart and kidney (Dehbi et al., 2010). The lung displayed the lowest Hsp‐72 expression. Variations in Hsp‐72 expression among organs were linked to the severity of organ damage, indicating varying susceptibility to heat‐induced injury (Dehbi et al., 2010). These findings align with previous rodent studies, which demonstrated similar tissue‐specific patterns of Hsp‐72 expression (Flanagan et al., 1995).

### 7.2 Extracellular HSPs

Recent research in humans with heat stroke has shown that HSPs, initially considered strictly intracellular, can be found in the circulation and act as danger signals by interacting with Toll‐like receptors (TLRs) eliciting an inflammatory response (Huisse et al., 2008). We observed a significant increase in circulating HSP72 levels in baboons with severe heat stroke, which remained elevated for up to 72 h post‐onset (Dehbi et al., 2010). Notably, circulating HSP72 levels correlated with markers of tissue damage in multiple organs (Dehbi et al., 2010). Elevated levels of circulating HSP70 were also documented in humans with classic heat stroke (Huisse et al., 2008). Together, this suggests that HSP72 may serve as a biomarker for organ injury, highlighting its potential diagnostic and prognostic value in heat stroke. More research is needed to investigate the mechanistic role of HSP72 in heat stroke pathogenesis and its clinical utility. Collectively, this raises intriguing questions about the role of the heat shock response in multiorgan injury and dysfunction during heat stroke.

## 8 THERAPEUTIC INTERVENTIONS AND LESSONS LEARNED

Studies in the baboon model have yielded valuable insights into therapeutic interventions for heat stroke (Bouchama, Kwaasi, et al., 2007; Bouchama et al., 2011, 2008). We evaluated activated protein C, a therapeutic agent known for its multifaceted properties, including anticoagulant, anti‐inflammatory, antiapoptotic and endothelial barrier stabilization effects (Looney & Matthay, 2006). The results indicated several advantages in baboons with heat stroke, such as attenuating the inflammatory response, decreasing endothelial cell injury and promoting cell survival (Bouchama et al., 2008). However, it did not effectively mitigate coagulation activation or lead to significant improvements in organ function or survival rates. We also examined dexamethasone, a frequently used anti‐inflammatory drug. Surprisingly, it exacerbated organ injury in the baboon model of heat stroke (Bouchama, Kwaasi, et al., 2007) in contrast to its neuroprotective effect observed in rodent models (Liu et al., 2000). Another therapeutic approach explored was recombinant nematode anticoagulant protein c2 (rNAPc2). It successfully blocked coagulation activation (Bouchama et al., 2011), but it did not lead to noticeable improvements in organ function or outcomes. This suggests that coagulopathy may not always be a prerequisite for severe organ dysfunction and mortality in heat stroke cases.

Overall, these findings underscore the complexity of the inflammatory and coagulation responses in heat stroke and emphasize the need for caution when considering anti‐inflammatory or anticoagulant agents in its treatment. It becomes evident that targeting a single pathway may not always yield benefits and could even be detrimental. Therefore, future research should explore therapies that simultaneously address multiple pathogenic pathways, such as recombinant activated protein C, to achieve more favourable outcomes in heat stroke treatment.

## 9 LIMITATIONS

While our study using the baboon model has provided valuable insights into the cellular and pathological aspects of heat stroke, certain limitations must be acknowledged. Baboons share many similarities with humans but also exhibit inherent differences that should be taken into account when extrapolating findings to humans. Baboons, exhibit distinct differences in organ function that could notably affect the pharmacokinetics and pharmacodynamics of drugs, impacting the translational applicability of our findings (Mueller et al., 2011). Also, variances in metabolic rates between baboons and humans may influence thermoregulatory responses and resilience to heat‐induced stress (Casiraghi et al., 2013).

Moreover, the immune response, a pivotal aspect in the pathophysiology of heat stroke, shows different patterns between humans and baboons, potentially leading to variations in inflammatory and repair mechanisms post‐heat stroke (Hawash et al., 2021). These species‐specific differences in immunity are crucial when considering the translation of our findings to human conditions.

### 9.1 Classic versus exertional heat stroke

Both classic and exertional heat stroke share fundamental pathophysiological processes such as hyperthermia, multiorgan injury, and activation of inflammatory and coagulation pathways, but there are critical differences (Bouchama et al., 2022). The key distinction lies in the role of muscle in exertional heat stroke, leading to pronounced rhabdomyolysis, acute kidney injury and lactic acidosis (Laitano et al., 2021). Muscles, acting as endocrine organs in exertional heat stroke, secrete bioactive molecules that significantly influence the inflammatory response and recovery processes (Laitano et al., 2021). Therefore, whereas insights from our baboon model of classic heat stroke are valuable, the direct applicability of these findings to exertional heat stroke is limited due to these distinct muscle‐related pathophysiological mechanisms.

## 10 CONCLUSION

The baboon heat stroke model has significantly advanced our fundamental understanding of the complex mechanisms underlying organ damage, which are challenging to study directly in humans. It has also paved the way for the development of more effective countermeasures against this life‐threatening condition. Continued utilization of this model for unravelling the pathophysiology of heat stroke and assessing novel treatments remains indispensable. With the growing threat of heat waves worldwide, ongoing research in this direction is crucial for advancing our ability to prevent and mitigate the impact of heat stroke on human health.

## AUTHOR CONTRIBUTIONS

Abderrezak Bouchama developed the baboon heat stroke model and designed most of the studies using this model, and contributed to writing and editing of the manuscript. George Roberts performed all histopathological parts of the studies using the baboon model of heat stroke. Mashan Abdullah and Salleh Ehaideb compiled all the data, reviewed and examined all the studies, retrieved and synthesized the findings, and contributed to the writing of the manuscript. All authors have read and approved the final version of this manuscript and agree to be accountable for all aspects of the work in ensuring that questions related to the accuracy or integrity of any part of the work are appropriately investigated and resolved. All persons designated as authors qualify for authorship, and all those who qualify for authorship are listed.

## CONFLICT OF INTEREST

The authors declare that they have no competing interests.

## FUNDING INFORMATION

No funding was received for this work.

## Supporting information

Supplemental Table 1. Thermal responses in baboons subjected to heat stress.
Supplemental Table 2. Biomarkers of organ function, inflammation, coagulation and stress response in moderate and severe heatstroke in baboons.
