# Supplementary material for: Insights into pathophysiology and therapeutic strategies for heat stroke: Lessons from a baboon model
Source: Exp Physiol. 2023 Dec 20;109(4):484–501. doi: 10.1113/EP091586 (PMC10988686; doi:10.1113/EP091586)
Supplement: Supplementary file 1 — Supplemental Table 1. Thermal responses in baboons subjected to heat stress. Supplemental Table 2. Biomarkers of organ function, inflammation, coagulation and stress response in moderate and severe heatstroke in baboons. [file EPH-109-484-s001.docx]

**Supplemental table 1. Thermal responses in baboons subjected to heat stress**

| Heat Response | Sham-Heated Control | Moderate Heatstroke | Severe Heatstroke |
| --- | --- | --- | --- |
| Weight, kg | 4.5 ± 0.2 | 4.2 ± 0.4 | 4.4-4.76 |
| Incubator temperature, °C | 27.7 ± 0.5 | 44.2 ± 0.9 | 44-47 |
| Incubator humidity, % | 36 ± 3.1 | 35.4 ± 1.7 | 35-37 |
| Duration of heat exposure, min | 267 ± 52 | 303 ± 61 | 245-494 |
| Tc maximum, °C | 36.5 ± 0.3 | 42.5 ± 0.0 | 43.4-43.6** |
| Heat load, °C/min | 0 | 249 ± 43.6 | 269-519 |
| Heating rate, °C/min | 0 | 0.019 ± 0.003 | 0.019-0.26 |
| Time at >40.4°C, min | 0 | 155 ± 47 | 157-315 |
| Cooling rate, °C/min | 0 | 0.056 ± 0.005 | 0.003-0.05 |

* Adapted from reference (Bouchama *et al.*, 2005).

** Tc Max was significantly different between moderate and severe heat stroke compared by Student’s t-test. Sham-heated control animals are shown for indication.

Heat stroke was induced by exposing the baboons to preset high environmental heat within a modified neonatal incubator. Sham heated animals in the same neonatal incubator preset at a lower environmental temperature for an equivalent time of the study groups served as a control.

Tc: core temperature was monitored by a pediatric rectal thermistor probe calibrated for 0–70°C with a precision of ± 0.15°C.

Heat load (°C/min) was calculated as Ʃ time interval (min) [Tc (°C) above 40.4°C - 40.4°C].

**Supplemental table 2. Biomarkers of organ function, inflammation, coagulation and stress response in moderate and severe heatstroke in baboons**

| Variables | Markers | Alterations/Findings | |
| --- | --- | --- | --- |
|  |  | **Moderate Heatstroke** | **Severe Heatstroke** |
| Electrolytes |  |  |  |
|  | Na^+^ | Mild increase | Moderate increase |
|  | Cl^-^ | Mild increase | Moderate increase |
|  | K^+^ | Normal or mild decrease | Normal or mild increase |
|  | HCO3^-^ | Mild decrease | Moderate decrease |
| Blood Sugar |  | Mild decrease | Moderate decrease |
| Organ Dysfunction |  |  |  |
| Acute kidney injury | Creatinine, Urea | No significant increase | Increased |
| Liver injury | ALT, AST, LDH | No significant increase | Elevated |
| Rhabdomyolysis | CK | Elevated | Elevated |
| Myocardial injury | Troponin |  | Increased |
| Disseminated intravascular coagulation (DIC) | Prothrombin Time (PT) | Increased | Prolonged |
|  | activated Partial Thromboplastin Time (aPTT) | No significant increase | Prolonged |
|  | Platelets | No significant decrease | Decreased |
|  | D-dimer | Increased | Elevated |
| Endothelial cell injury | Thrombomodulin | No significant increase | Increased |
| Inflammation | Leukocyte | Leukocytosis | Leukopenia |
|  | Pro-inflammatory cytokine (IL-6)  Anti-inflammatory cytokine (IL-10)  Chemokine (IL-8)  Soluble cytokine receptor (IL-1ra, sTNFr1, sTNFrII) | Mild increase  Mild increase  Mild increase  Mild increase | Marked increase  Marked increase  Marked increase  Marked increase |
|  | Regulatory cytokine (IL-12p40) | Decreased | Decreased |
| Coagulation and Fibrinolysis | **Coagulation**  TAT  Protein C  Protein S  Antithrombin | NA  NA  NA | Increased  Decreased Decreased  Decreased |
|  | **Fibrinolysis**  PAP  tPA  PAI | NA  NA  NA | Increased  Increased  Decreased than increased |
| Heatstress response | eHSP72 | NA | Increased |
|  |  |  |  |

Bouchama A, Roberts G, Al Mohanna F, El-Sayed R, Lach B, Chollet-Martin S, Ollivier V, Al Baradei R, Loualich A, Nakeeb S, Eldali A & de Prost D (2005). Inflammatory, hemostatic, and clinical changes in a baboon experimental model for heatstroke. *J Appl Physiol* **98,** 697-705.
